# Discrimination between normal and cancer white blood cells using holographic projection technique

**DOI:** 10.1371/journal.pone.0276239

**Published:** 2022-10-20

**Authors:** Rania M. Abdelazeem, Dahi Ghareab Abdelsalam Ibrahim

**Affiliations:** 1 Engineering Applications of Laser Department, National Institute of Laser Enhanced Sciences “NILES”, Cairo University, Giza, Egypt; 2 Engineering and Surface Metrology Laboratory, National Institute of Standards, Giza, Egypt; The Ohio State University, UNITED STATES

## Abstract

White blood cells (WBCs) play a vital role in the diagnosis of many blood diseases. Such diagnosis is based on the morphological analysis of blood microscopic images which is performed manually by skilled hematologist. However, this method has many drawbacks, such as the dependence on the hematologist’s skill, slow performance, and varying accuracy. Therefore, in the current study, a new optical method for discrimination between normal and cancer WBCs of peripheral blood film (PBF) images is presented. This method is based on holographic projection technique which is able to provide an accurate and fast optical reconstruction method of WBCs floating in the air. Besides, it can provide a 3D visualization map of one WBC with its characterization parameters from only a single 2D hologram. To achieve that, at first, WBCs are accurately segmented from the microscopic PBF images using a developed in-house MATLAB code. Then, their associated phase computer-generated holograms (CGHs) are calculated using the well-known iterative Fourier transform algorithm (IFTA). Within the utilized algorithm, a speckle noise reduction technique, based on temporal multiplexing of spatial frequencies, is applied to minimize the speckle noise across the reconstruction plane. Additionally, a special hologram modulation is added to the calculated holograms to provide a 3D visualization map of one WBC, and discriminate normal and cancer WBCs. Finally, the calculated phase-holograms are uploaded on a phase-only spatial light modulator (SLM) for optical reconstruction. The optical reconstruction of such phase-holograms yields precise representation of normal and cancer WBCs. Moreover, a 3D visualization map of one WBC with its characterization parameters is provided. Therefore, the proposed technique can be used as a valuable tool for interpretation and analysis of WBCs, this in turn could provide an improvement in diagnosis and prognosis of blood diseases.

## 1- Introduction

**Fig 1:**
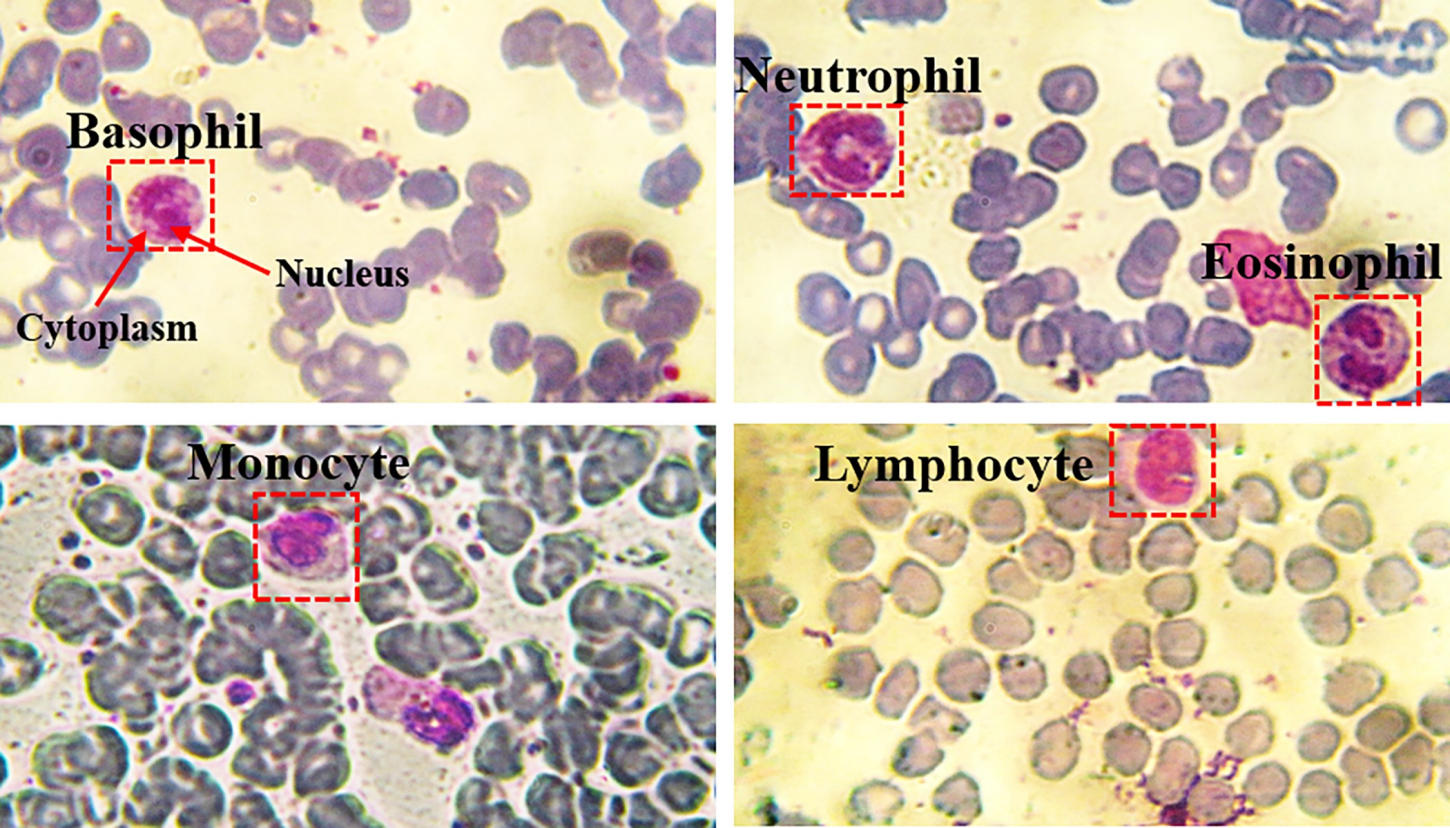
The five different kinds of WBCs in PBF images of normal blood samples with a magnification of 60X. Note that, the depicted images are captured at National Institute of Laser Enhanced Science “NILES”, Cairo University.

Classical optical microscopy is a common monitoring and diagnostic technique for characterizing different blood diseases and evaluating their progression. It is based on counting and classifying white blood cells (WBCs) because they play a central role in our immune system. WBCs are the effector cells that circulate across the lymphatic system and the bloodstream [1, 2]. A peripheral blood film (PBF) is a comprehensive qualitative examination tool that is significantly utilized to detect abnormalities in WBCs. It is made from a fresh drop of blood from a finger-stick puncture or a syringe. The blood drop is placed on one side of a glass slide and then is smeared to produce a blood film of length about 2.5 cm [3]. Generally, WBCs are categorized into five categories: Basophils, Eosinophils, Lymphocytes, Monocytes, and Neutrophils [3–5], as shown in Fig 1. After staining the PBF with Romanowsky-type stains, the nucleus has a dark purple appearance while the cytoplasm appears with a pink [3, 6], demonstrated in Fig 1. The five categories appear to have different morphology, cell size, and nucleus size. Each specific kind of WBCs is responsible for a specific function in the immune system [7]. Monocytes are the largest WBCs (diameter 12–20 μm) that are derived from the bone marrow, it is responsible for regulation of cellular homeostasis in case of infection. Lymphocytes are divided into two kinds: B and T, and they are responsible for defense against foreign organisms such as viruses. Neutrophils are commonly involved for defense against bacteria. Basophils and Eosinophils are responsible for the defense against parasites, allergy, and autoimmune diseases. The approximate number of WBCs in a normal peripheral blood film is between 4,000 to 10,000 cells/microliter, most of them are neutrophils. In contrast, for a cancer PBF the number of WBCs ranges from 100,000 to 400,000 cells/microliter.

**Fig 2:**
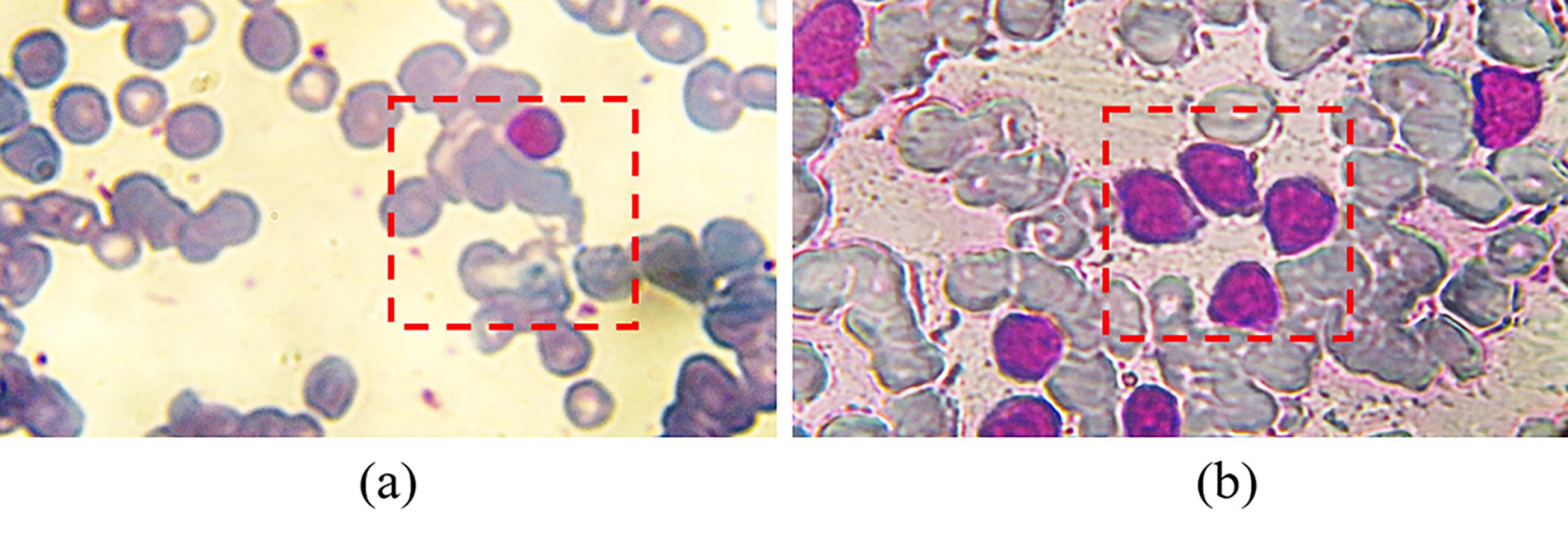
PBF images: (a) normal, and (b) cancer blood samples with a magnification of 60X. Note that, the depicted images are captured at NILES, Cairo University.

WBCs act as defenders of the body against infection and diseases by destroying the germs and the viruses. Accordingly, the change of the number of WBCs in the blood indicates the existence of specific disease [1, 8–12]. Thereby, differential count of WBCs can lead to diagnose of a specific disease in an early stage. According to the disease kind, the number of WBCs can either decrease (called leukopenia) or increase (called leukocytosis) [13]. Fig 2 shows two PBF images of a normal blood sample (Fig 2(A)) and a cancer blood sample (Fig 2(B)), the number of Lymphocytes inside the red dashed box increased about four times in case of blood cancer and their size is changed as well.

There are two different approaches to count WBCs, an automatic approach and a manual approach. For the automatic approach, flow cytometry is commonly used to differentially count the five kinds of WBCs [14]. This method is more convenient for investigation of large volume blood samples. However, this method doesn’t capture any cell images so it couldn’t provide any morphological information about WBCs in addition to its expensive price. While, the manual approach is applied on a PBF utilizing the classical microscope system. Where, the PBF sample can be viewed directly by the microscope eyepiece or it can be photographed. Within this approach, the monolayer regions are scanned by mechanical micrometer to count the total number of WBCs. This method is more commonly used in laboratories and hospitals than the automatic approach [8, 9, 11, 13]. However, this approach is a qualitative-based because it is based in its calculations on two-dimensional (2D) morphological shape of the WBCs. Additionally, using such a qualitative technique leads to inter-observer variability. Moreover, it gives approximate number of total WBCs and it doesn’t give direct information about cell morphology and structure.

The judgment criteria for discrimination between normal and cancer PBF is based on specific factors. First, the number (as previously mentioned) ranges from 100,000 to 400,000 cells/microliter for a cancer PBF. Second, the cancer WBCs are commonly associated with Lymphocyte type of WBCs. Finally, the morphology (size and shape) of Lymphocyte cells are dramatically different. That is, cancer Lymphocyte WBCs are larger than that of the normal WBCs as depicted in Fig 2(B). Besides, the cancer Lymphocyte WBCs often have abnormal shape (rather than uniform circular shape) and their nuclei appear larger than normal cells and with an irregular shape. In the current study, similar judgement criteria have been followed and verified using holographic projection technique.

Holographic projection is considered as one of the most promising techniques for reconstructing high resolution 3D information of an object. From the medical point of view, it is regarded as an encouraging tool for reconstructing precise representation of organs’ anatomical structure [15–18]. Computer-generated holograms (CGHs) allow direct access to both the intensity and the phase of an object to provide a complete 3D information of it [19]. Although the calculated CGH is a 2D distribution, it encodes complete 3D information of an object.

Moreover, it aids the physicians for better understanding and analysis of different body organs [20–23]. Accordingly, it has a great impact on diagnosis of various diseases, surgical planning, and minimizing the invasive surgeries.

To the best of authors knowledge, the present study proposes a new optical method based on holographic projection to visualize and discriminate normal and cancer WBCs, and to highlight their morphology and structure as well. The great advantage of such discrimination method over the commonly used methods in literature such as pattern recognition is the ease of computation and optical reconstruction of holograms where both are executed in real-time. Moreover, large amount of information can be encoded in only a single 2D hologram without the loss of the information. To achieve the goal of the study, at first, the normal and cancer WBCs are precisely segmented from microscopic PBF images using a developed in-house MATLAB code. Then, their associated phase-holograms are calculated using iterative-Fourier transform algorithm (IFTA). Within this approach, a speckle noise reduction algorithm is applied to minimize the speckle noise across the observation plane. Additionally, a special hologram manipulation is added to the calculated holograms to provide a 3D visualization map of one WBC using a single 2D hologram and to discriminate two WBCs (normal and cancer cells). Finally, the calculated holograms are uploaded on a reflective phase-only SLM for optical reconstruction.

## 2- Samples preparation and WBCs segmentation process

### 2.1 PBF samples collection

The employed PBF samples in this framework were prepared at Al-Kasr Al-Aini hospital, Cairo University. The protocol of the investigated samples which were taken from two volunteers, has been reviewed by “NILES’ Ethics Committee, Cairo University” who gave it the approval number of “Cu–NILES / 21 / 2”. The volunteers provided their written informed consents to use their samples in this work. The PBF images were acquired at NILES, Cairo University, using a phase contrast microscope (LABOMED Europe GmbH, Model: TCM 400) operated at the bright field mode with magnification of 60X. Since the objective of this study is to investigate the morphology and structure of the WBCs, we propose to utilize an accurate segmentation process based on an in-house developed MATLAB code.

### 2.2 The proposed segmentation method

There are different approaches for the segmentation process. For instance, graph-based, clustering, region growing, and thresholding segmentation are the common approaches. In the present paper, the thresholding approach is used for extracting the WBCs from the microscope images. The beginning of the segmentation process is to obtain the red, green, and blue components (RGB) of the colored raw microscope images of the cells. It was revealed that the green component of the images exhibits highest contrast and sharpness amongst the three components. Then, the histogram of the green image component was adjusted to further increase the contrast between the targeted WBC and the image background. In order to allow low computational cost, the targeted WBC was extracted from the microscope green component image by cropping the images. The resultant cell image is converted to a binary image using a threshold of 70% of the bright area intensity (I_B_), where the white pixels have a value of 1 while the black pixels have a value of 0. The unwanted dots or regions of white pixels are removed from the image. That is, the removed regions are extracted from the image such that the regions of which the connected white pixels are less than a certain number (i.e., less than 50000 pixels). The holes (black pixels) inside the targeted region is filled. Then, a gaussian smoothing filter of a window size of 15 and a threshold of 50% were used for smoothing the edges before starting the edge detection. The edge detection process is conducted using a Sobel filter of G_x_ and G_y_ of [-1 0 1; -2 0 2; -1 0 1] and [1 2 1; 0 0 0; -1–2–1], respectively.

**Fig 3:**
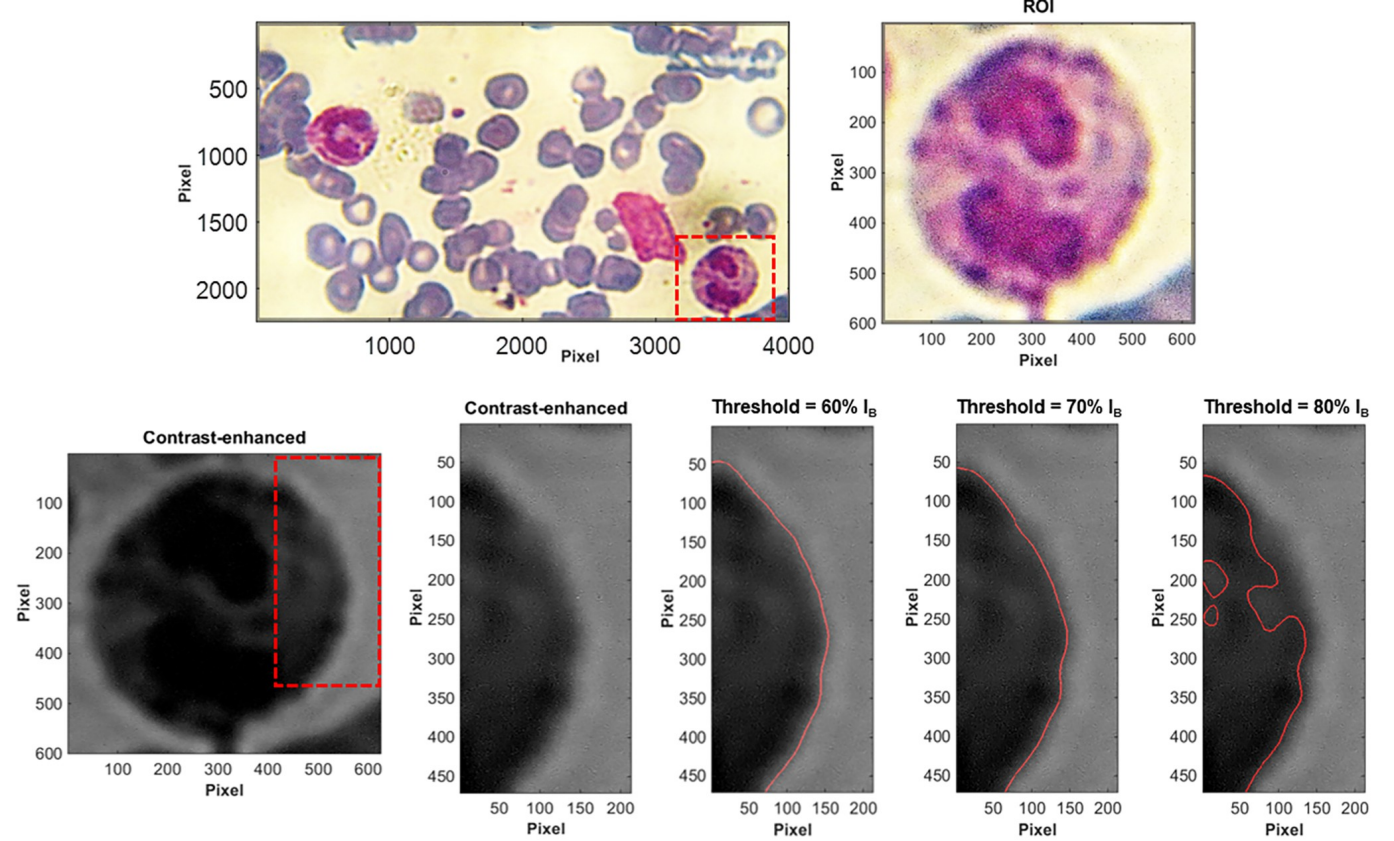
Demonstration of the influence of the threshold in the image processing procedure.

The influence of different threshold values is demonstrated in Fig 3. The figure shows a selected region of interest (ROI) containing eosinophil WBC and its corresponding contrast-enhanced green component. The result reveals that a threshold value of 70% I_B_ satisfies most of the tested cases (see Fig 3). It should be noted that at a threshold higher that 70% I_B_, the segmentation process starts to discard necessary regions of the actual WBC. On the other hand, at a threshold below 60% I_B_, the segmentation process mistakenly considers a larger area than the correct area of the WBC (not shown here).

### 2.3 Evaluation of the segmentation process

In order to evaluate the accuracy of the proposed segmentation method, our results are compared with the results obtained by the open-source cell image analysis software “CellProfiler^TM^ [24–27]”. This software is commonly used to identify, count, and measure different biological objects in an image. Besides, it provides a specific object’s features including, its size, location, shape, color, texture (smoothness), and the number of its neighbors. To achieve the evaluation process, a new pipeline is created, which is a sequential set of image analysis modules, in order to segment the nucleus of Neutrophil cell (as an example) from a normal blood microscope image. At first, the image is dragged into the program, named, and its type was selected by the module “Names And Types”. Next, “Color To Gray” module was added to the pipeline to split the color microscope image into its gray level components (R, G, B). Then, the image analysis module “Identify Primary Objects” is added to the pipeline. This module is used to identify the objects without depending on any information other than an input gray scale image, i.e., the nuclei are typically primary objects. Therefore, the edges of the nucleus are determined by this module from the green component image by selecting the typical diameter of the objects to be identified to be between 10 and 40 pixels using minimum cross-entropy threshold method with smoothing scale of 1.3488. Another module “Threshold” was added to the pipeline where the threshold strategy was set to global using minimum cross-entropy threshold method with lower and upper bounds of 0.3 and 0.41. The threshold image is smoothed by a median filter using “Smooth” module. The image processing module “Enhance Edges” was added to the pipeline and applied to the smoothed image to enhance the detected edges by using Sobel Operator. Finally, the segmented nucleus image is exported by adding “Save Images” module to the pipeline.

**Fig 4:**
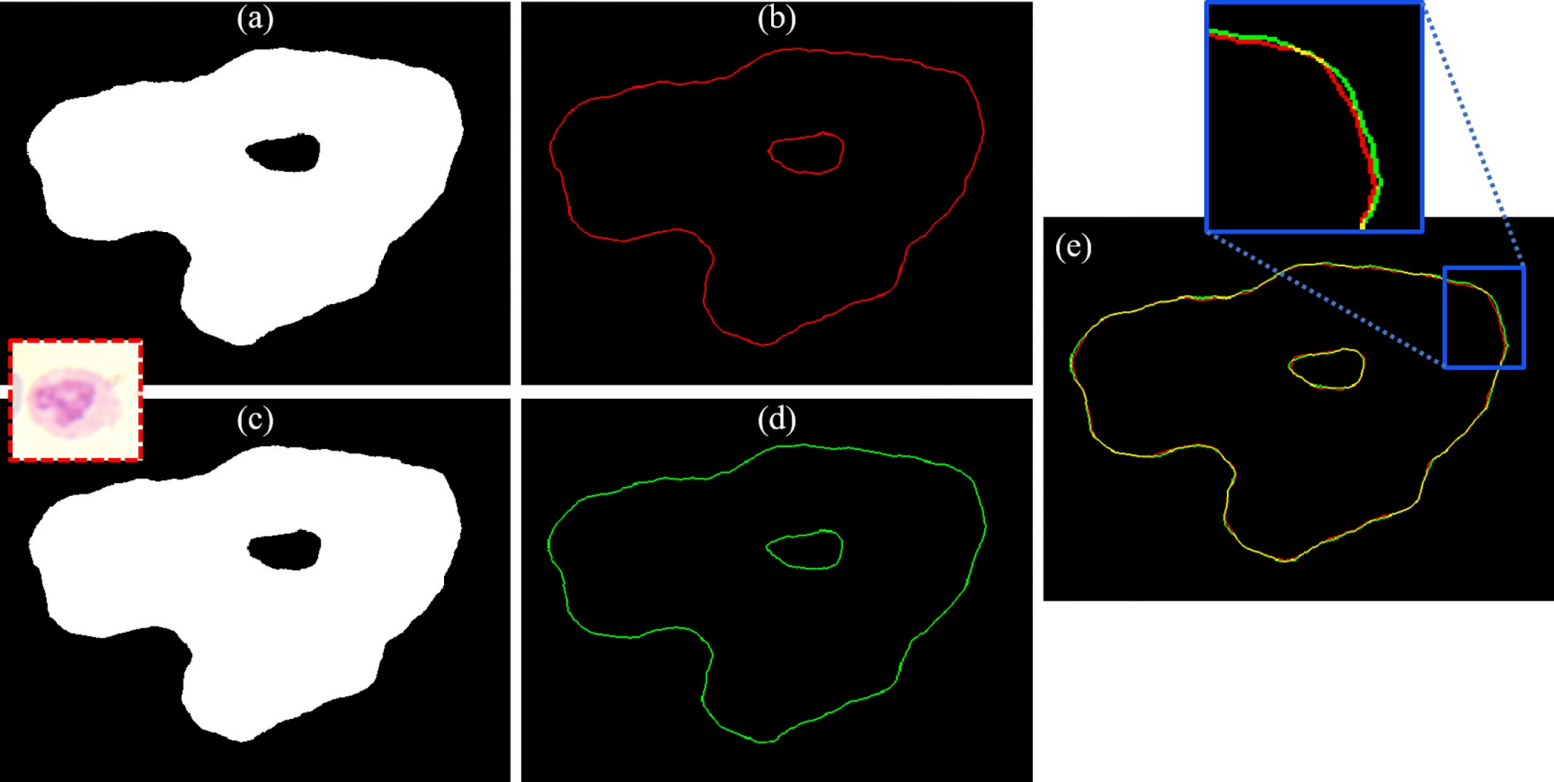
The segmentation results of the nucleus of Neutrophil WBC: (a) and (b) the segmented nucleus and its edges that are obtained by CellProfiler^TM^ software, (c) and (d) the segmented nucleus and its edges obtained by our proposed segmentation method, and (e) the overlapping between the detected edges from the two methods.

Fig 4(A) and 4(B) show the segmentation results of the nucleus and the edges of Neutrophil WBC by CellProfiler^TM^ software. While, the results obtained by our proposed segmentation method are presented in Fig 4(C) and 4(D). The overlapping of the detected edges from both methods is shown in Fig 4(E). To calculate the segmentation accuracy, Jaccard similarity coefficient which is used to compute the intersection between our segmented nucleus and the segmented nucleus which is obtained by CellProfiler^TM^ was used. The calculated similarity was 0.9853 which indicates an extremely high overlap between both results.

## 3- Phase-only holograms generation

**Fig 5:**
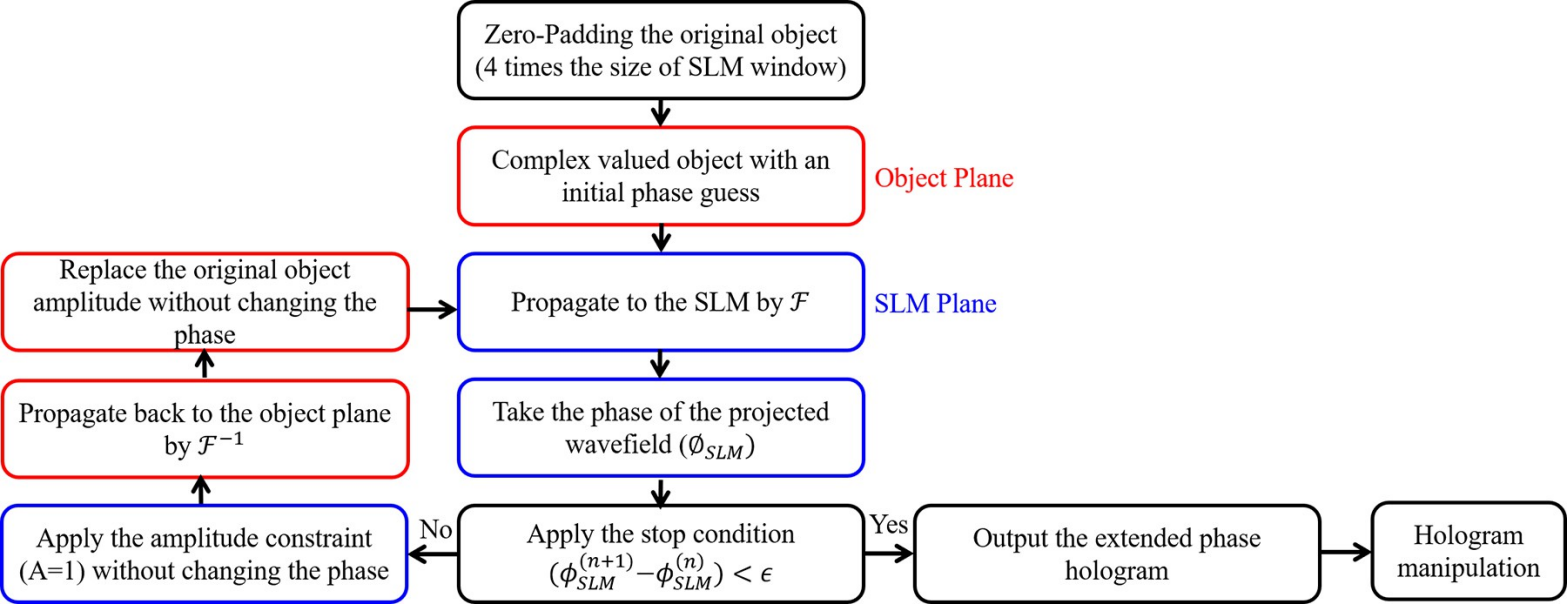
Flowchart of the iterative Fourier transform algorithm (IFTA) where $F\;and\;F^{-1}$ denote fast Fourier transform and inverse fast Fourier transform, ∅_*SLM*_ is the phase of the wavefield at the SLM plane, and *n* is the iteration number.

The computer-generated holograms (CGHs) of the normal and cancer WBCs are calculated and optimized using the well-known iterative Fourier transform algorithm (IFTA) [2, 28]. The flowchart of the iterative algorithm is depicted in Fig 5. The algorithm considers two parallel planes, one is the object/reconstruction where the object is placed and the other is the SLM plane where the CGH is generated. The projection which is used to propagate the wavefield from one plane to the other and back again to the first plane consists of Fourier transformation (F) for forward propagation and inverse Fourier transformation ${(F}^{-1})$ for backward propagation. At each plane an intensity constraint is applied. At the SLM plane, it is assumed a plane wave illumination which means that the amplitude is homogeneous and equal 1 everywhere. At the object plane, the original object amplitude, that is calculated by taking the square root of the intensity, replaces the obtained one after the implementation of $F^{-1}$. At the SLM plane, another amplitude constraint is applied (a plane wave illumination, i.e., *A* = 1), while the phase of the propagated wave field ($\phi_{SLM}^{\left(n\right)}$) is kept unaffected.

The iterative process is repeated until no change in the phase is observed ${(\phi }_{SLM}^{\left(n+1\right)}-\phi_{SLM}^{\left(n\right)})<\epsilon$, where *ϵ* is the phase difference at the convergence of the algorithm. This is monitored through the numerical reconstruction by calculating the root mean square error (RMSE) which estimate the difference between the input image (reference image) and the image results from the numerical reconstruction (numerically reconstructed image), and it is calculated by [29]:

$$RMSE=\sqrt{mean{\left|\left(I_{Ref}-I_{NR}\right)\right|}^{2}},$$ (1)

where *I*_*Ref*_ and *I*_*NR*_ are the reference image and numerically reconstructed image.

**Fig 6:**
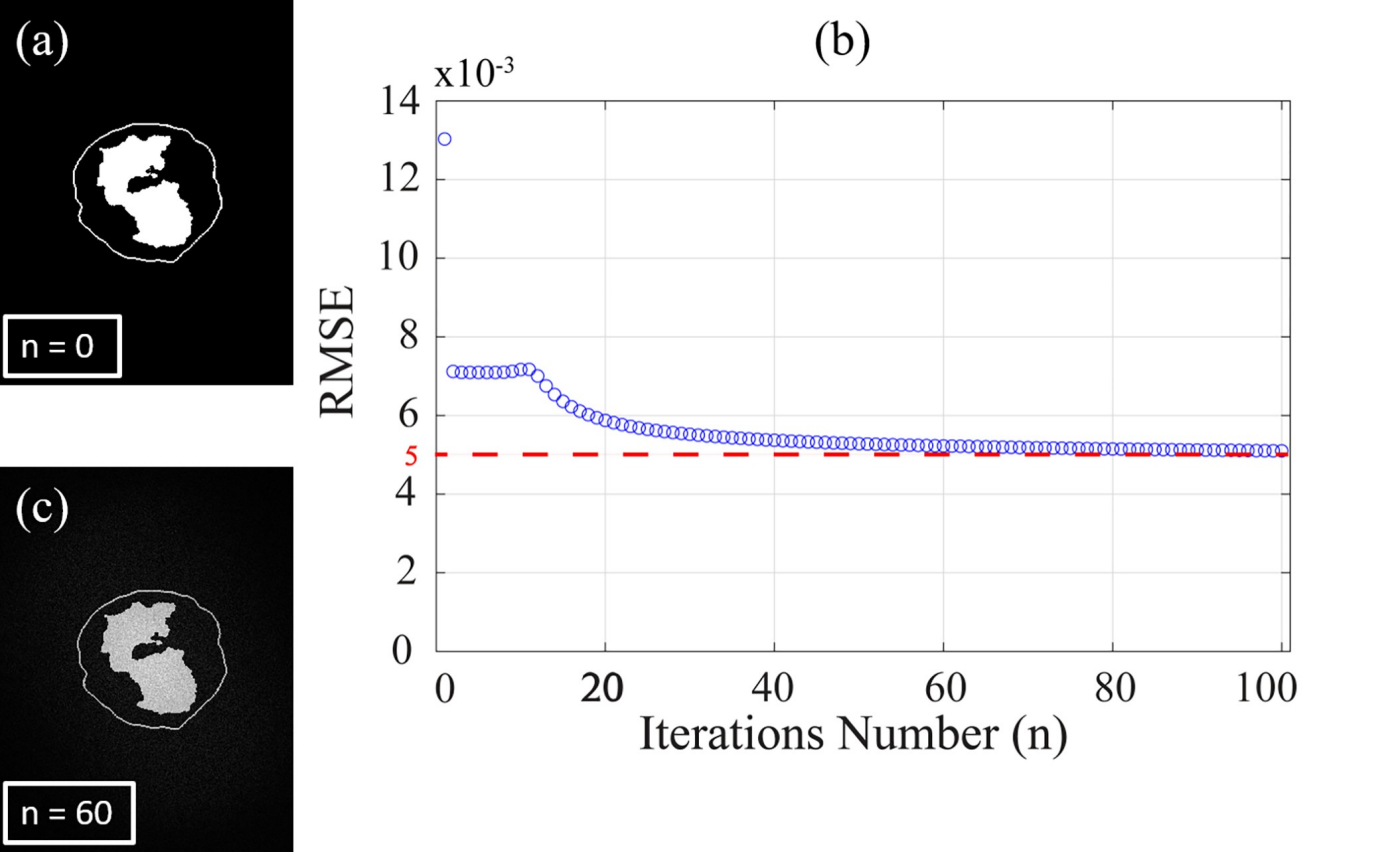
(a) Zero-padded Basophil reference image (image size = 7680 ×4320 pixels), (b) relation between number of iterations (n) and the root mean square error (RMSE), and (c) the numerical reconstruction of Basophil WBC image at n = 60.

Consequently, RMSE is used as a stopping criterion for the IFTA when the condition of the phase difference of the convergence is less than a predefined value (*ϵ*). As an example of calculating and optimizing the CGH using IFTA, the Basophil WBC is studied. The input of the algorithm is the zero-padded Basophil reference image (image size = 7680 ×4320 pixels) which is depicted in Fig 6(A). Note that, the reference image size is increased as a preprocessing step to reduce the speckle noise in the optical reconstruction of the phase holograms (this will be discussed later in Sec. 5). Fig 6(B) shows the relation between the RMSE and the number of iterations (n). It is clear from the figure that the RMSE decreases gradually by increasing the number of iterations (n), and it stabilizes at n = 60 to be 5×10^−3^ which corresponds to a phase difference of convergence ϵ ≤ 0.2 radians. The computation time that is required for designing the phase hologram (hologram size = 7680 ×4320 pixels) to achieve the convergence of the algorithm was 65.812 sec using Core i7-1165G7 CPU 2.8 GHz with 32 GB RAM, MATLAB 2018a. The numerically reconstructed Basophil image at n = 60 is shown in Fig 6(C).

Finally, the final phase hologram after convergence of the algorithm is displayed on a phase-only SLM for optical reconstruction. Illuminating the SLM with a plane wave generates WBC image floating in the air across the back focal plane of a Fourier lens.

### 3.1 Holograms manipulation

A special hologram manipulation is added to the calculated holograms to provide a 3D visualization map of a WBC form only a single 2D hologram. This method is able to segment and discriminate the cell or a portion of its structure (i.e., the cell, the edges of the cell with its nucleus, and the nucleus only). The manipulation of the calculated CGH is achieved by using three phase ramp (*R*_*z*_) and quadratic chirp functions (i.e., transfer function of propagation, *χ*_*z*_). These functions are pure phase functions and could be realized by the SLM. Therefore, the resultant CGH after manipulation is expressed as:

$$U_{SLM,m}=U_{SLM,m}^{n+1}.R_{z,m}.\chi_{z_{m}}$$ (2)

$$R_{z,m}=e^{ik[\sin\left(\alpha_{m}\right).\nu_{i}+\sin\left(\beta_{m}\right).\nu_{j}]}\&\chi_{z_{m}}=e^{\left[ik\frac{\lambda^{2}}{2}z_{m}{\left|\vec{\nu }\right|}^{2}\right]}$$ (3)

where m refers to the desired portion of the cell which will be visualized and segmented and it takes the values of m = (1, 2,3), *z*_*m*_ is the propagation distance between two portions of the cell, *k* is the wave number, $\vec{\nu }=(\nu_{i},\nu_{j})$ is a 2D vector across the SLM plane. The phase ramp is equivalent to tilt the SLM with angles of *α*/2 in *ν*_*i*_ direction and *β*/2 in *ν*_*j*_ direction, these two angles are defined by the distance between the SLM plane and the reconstruction plane and the required shift (${\Delta \vec{u}}_{m}$) to laterally separate the overlapping between the feature of interest and other features where sin(*α*_*m*_) = Δ*u*_*i*_/*z*_*m*_ and sin(*β*_*m*_) = Δ*u*_*j*_/*z*_*m*_. Finally, the complex modulated holograms are added to provide only a single 2D hologram where its phase is calculated to be displayed on the SLM for the optical reconstruction as:

$$\phi_{all}=arg(\sum_{1}^{3}U_{SLM,m})$$ (4)

## 4- Experimental verification

**Fig 7:**
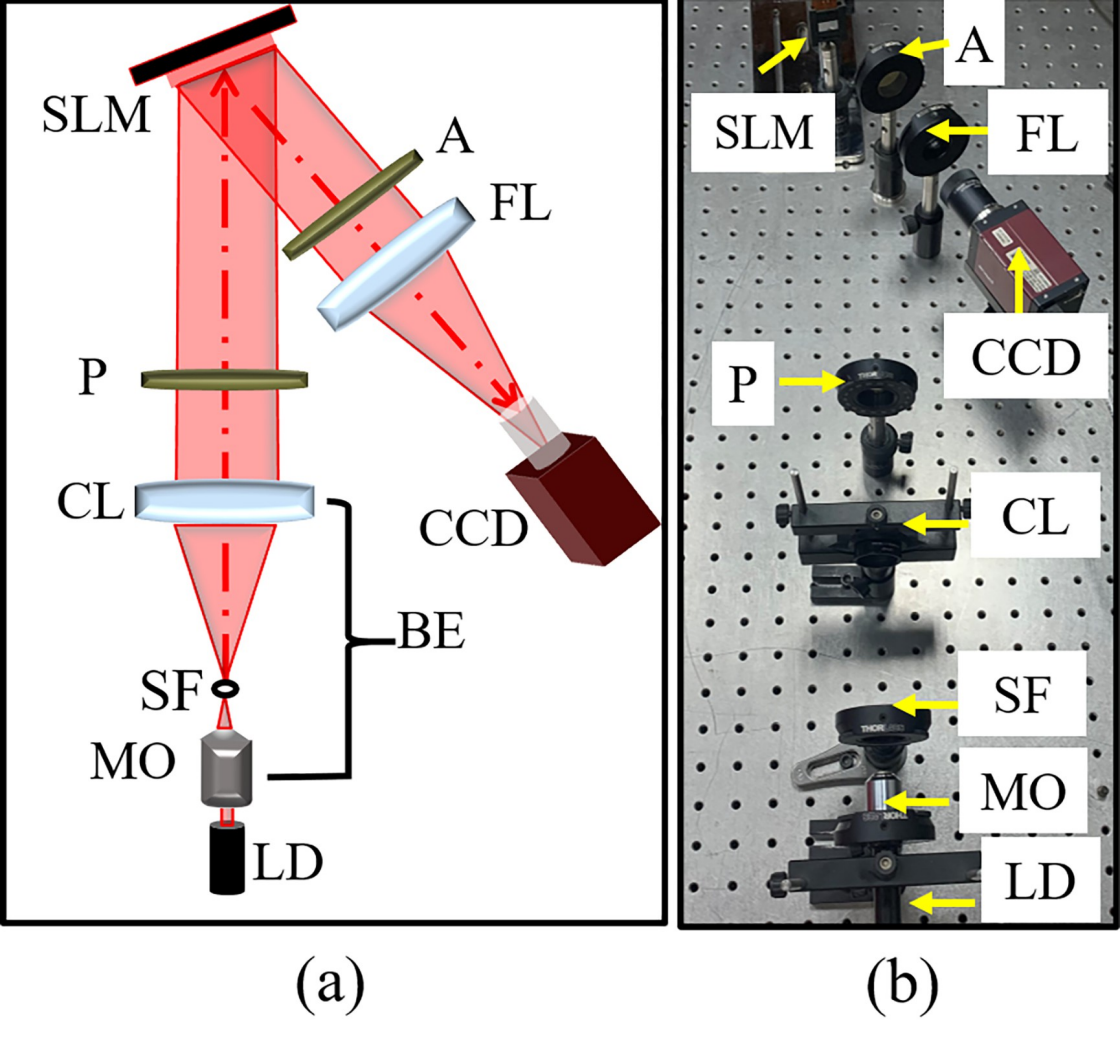
The proposed holographic projection system for the optical reconstruction of WBCs: (a) the schematic diagram and (b) a photograph of the experiment. LD: Laser diode; MO: microscope objective; SF: spatial filter; CL: collimating lens (f = 150 mm); BE: beam expander; P and A: polarizer and analyzer; SLM: spatial light modulator; FL: Fourier lens (f = 125 mm); CCD: charged coupled device.

Fig 7(A) shows a schematic layout of the optical setup of the proposed holographic projection system which is utilized for reconstructing WBCs. A red laser diode (LD) with a wavelength of (λ = 670 nm) is expanded and collimated by a beam expander (BE) to illuminate the SLM window (model: HOLOEYE PLUTO consists of a liquid crystal on silicon (LCOS), resolution: 1920×1080 pixels, pixel pitch: 8μm). A polarizer (P) and analyzer (A) are placed before and after the SLM window to select the correct polarization state with respect to the SLM’s slow axis (because the SLM is a birefringent modulator). SLM is a dynamic device that modulates the incident light according to the displayed pattern [30, 31], in analogy to the deformable mirror [32], to provide a predefined light intensity across the observation plane. It is placed at the front focal plane of a Fourier lens FL (f = 125 mm). A CCD camera (model: Pike F_505B, resolution: 2452×2054 pixels, pixel pitch: 3.35μm) is placed at the back focal plane of the Fourier lens FL to capture the projected intensity images of the WBCs. Fig 7(B) shows a photograph of the experimental setup.

## 5- Results and discussion

From the literature [29, 33–35], it is well-known that the optical reconstruction of the phase-only CGHs is noisy. To overcome the speckle noise which is a common problem in reconstructing phase-only CGHs, a speckle noise reduction algorithm which is based on temporal multiplexing of spatial frequencies is applied [33]. The algorithm is started by zero-padding the original WBC image to make its size four times the size of the SLM window (1920×1080 pixels). Then, the obtained zero-padded image which has the size of 7680 × 4320 pixels will be the input object to the IFTA. Consequently, the obtained extended phase hologram is divided into sixteen equal holograms each of them has a size of 1920×1080 pixels. The sixteen holograms are displayed sequentially on the phase-only SLM. The SLM modulates the incident plane wave according to the displayed phase pattern. Thus, each of the sixteen displayed patterns provides a projected intensity with different features of speckle. Therefore, the temporal multiplexing of the sixteen projected intensities yields a speckle-free reconstructed image across the observation plane. It is worth noting that, the time required to project the sixteen phase holograms and to capture the projected images is 0.8 sec, since the switch time of the utilized phase-only SLM is 50 msec.

To evaluate the validity and functional utility of the proposed holographic projection system, we estimated this throughout two levels. The first level includes the evaluation of both segmentation process and the designed CGHs. The segmentation process is evaluated based on estimating the optimum threshold value to segment the WBC from the microscope image and calculating the execution time of the segmentation process. It was found that the average execution time of the segmentation process was 5.35 sec using core TM i7-1165G7, CPU 2.8 GHz, 32 GB RAM, MATLAB 2018a which indicates that our proposed segmentation algorithm is accurate and fast. Then, the evaluation of the calculated CGHs is accomplished by calculating the difference between the input target WBC image (reference image) and the image that results from the numerical reconstruction of the calculated phase CGH (numerically reconstructed image after the convergence of the IFTA). This is achieved by calculating the RMSE which is based on measuring the difference between the reference image and the numerically reconstructed image to determine the optimum number of iterations (n) which is based on stopping criterion. It was found that at ϵ ≤ 0.2 radians, satisfying numerical reconstruction results are obtained with an average computation time of a phase-only hologram of 70.321 sec. While at the second level, the evaluation is based on the quantitative measurement of the quality of the optically reconstructed images. This is investigated by calculating the speckle noise contract (C), standard deviation (σ), signal to noise ratio (SNR). C is the ratio between the standard deviation (σ) of the optically reconstructed image by its mean value (μ). The scaled SNR is calculated by dividing the predefined intensity (reference WBC image) by the square norm of the difference between the reference intensity and the optically reconstructed intensity image as [33, 36]:

$$SNR=\frac{{\left\|I_{Ref}\right\|}^{2}}{{\left\|I_{Ref}-\beta I_{OR}\right\|}^{2}}$$ (5)

$$\beta^{2}=\frac{{\left\|I_{Ref}\right\|}^{2}}{{\left\|I_{OR}\right\|}^{2}}$$ (6)

here *I*_*Ref*_ and *I*_*OR*_ are the reference and the optically reconstructed images.

**Fig 8:**
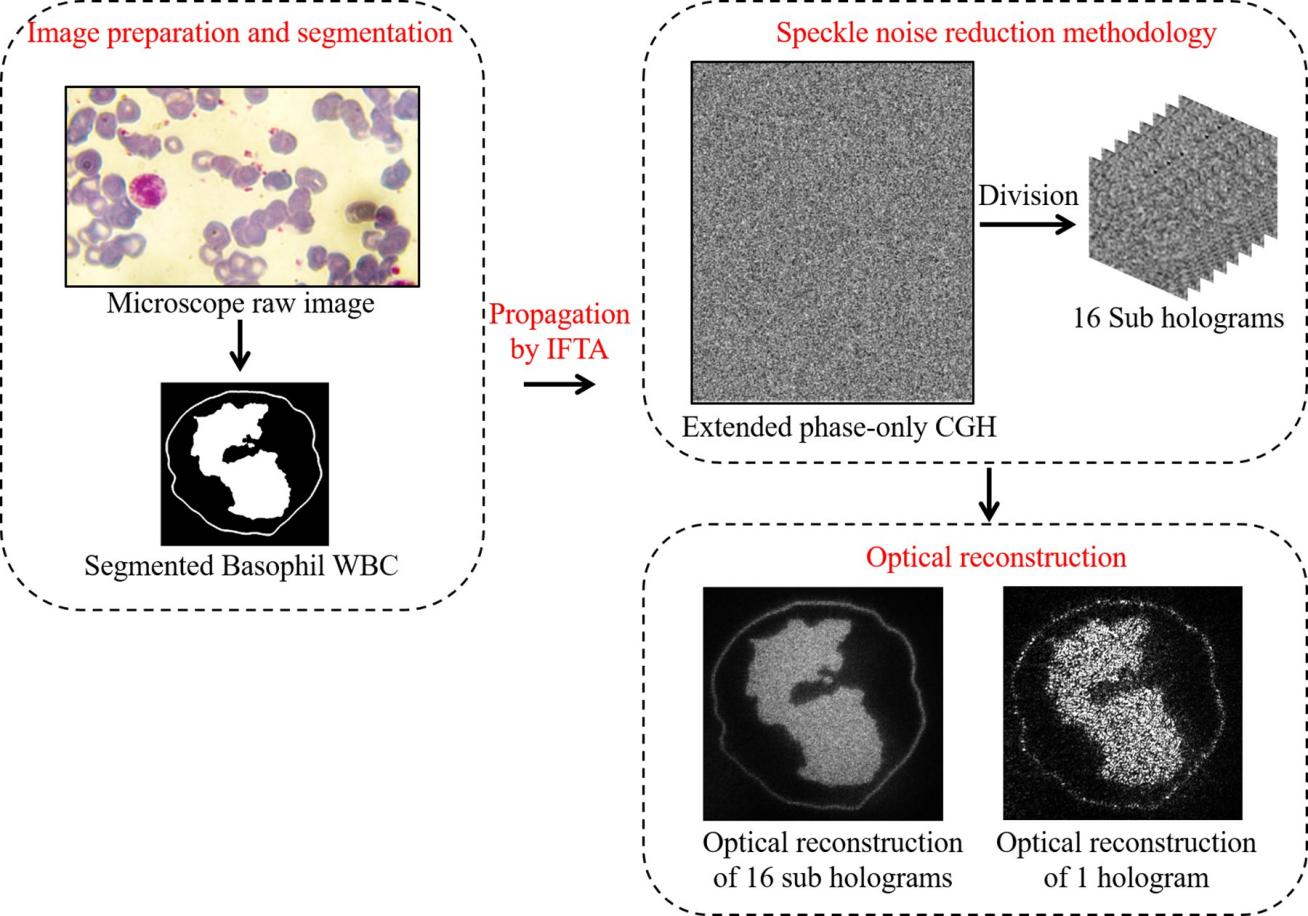
Flowchart summarizes the proposed work starting from the raw microscope image to the final optical reconstruction.

The overall process of segmentation of WBCs, holograms generation, modulation, speckle noise reduction methodology, and the optical reconstruction results is summarized in the flowchart depicted in Fig 8.

### 5.1 Segmentation results

**Fig 9:**
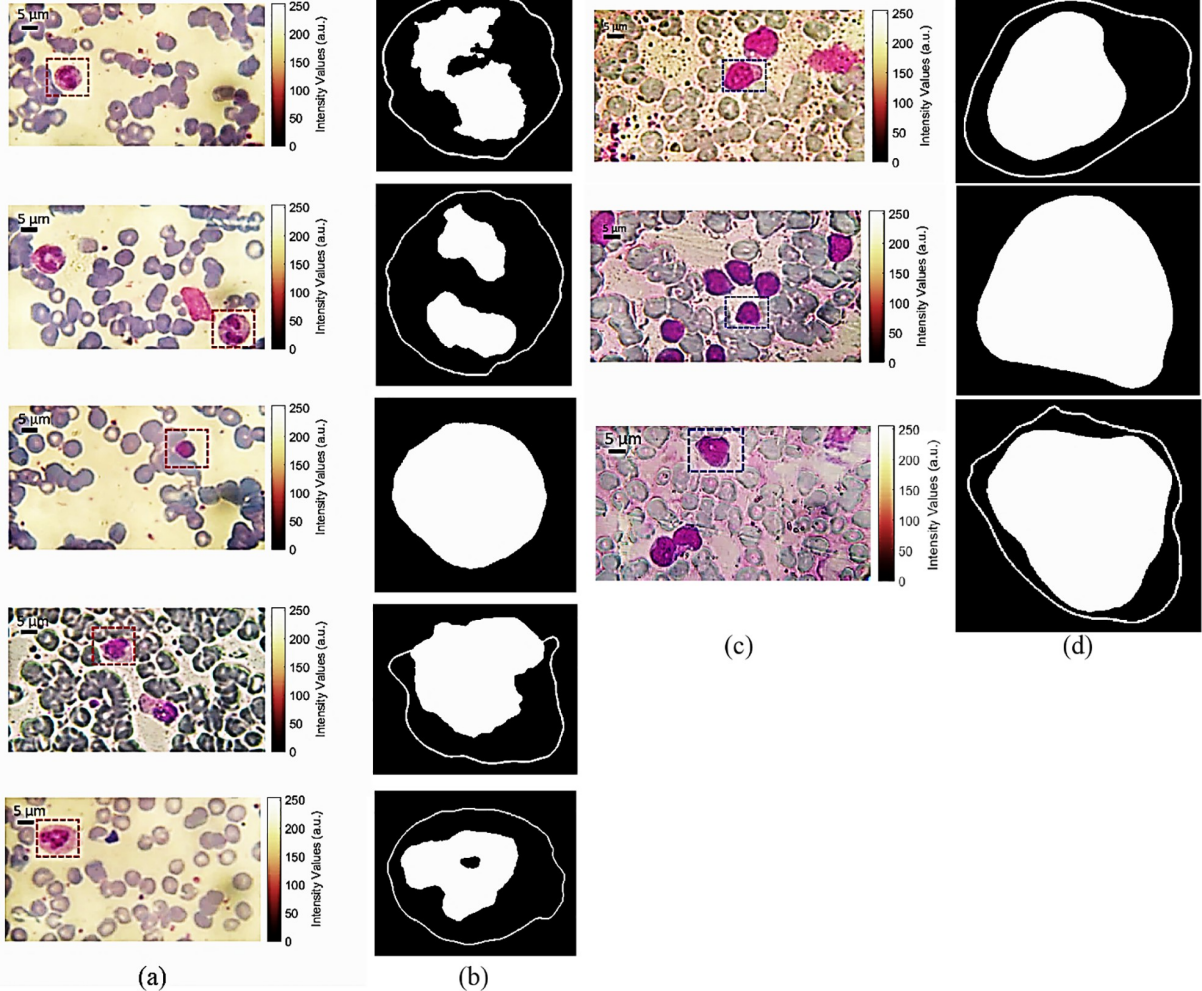
(a) and (c) optical microscope raw images of normal and cancer blood samples with a magnification of 60X, (b) segmentation results of the five kinds of normal WBCs (Basophil, Eosinophil, Lymphocyte, Monocyte, and Neutrophil, respectively), and (d) segmentation results of cancer Lymphocytes WBCS.

The results of the segmentation process for extracting the five kinds of normal WBCs (Basophil, Eosinophil, Lymphocyte, Monocyte, and Neutrophil, respectively) from the microscope images which are shown in Fig 9(A) are demonstrated in Fig 9(B). While, the segmentation results to extract the cancer Lymphocytes WBCs from the microscope images which are shown in Fig 9(C) are depicted in Fig 9(D). It is clear from the segmentation results, that the shape of the nucleus and its surrounding cytoplasm indicates a relevant information about the kind of the WBC. The nuclei of Basophil and Eosinophil WBCs have 2 segments, while for Neutrophil the nucleus has 4 segments. The nucleus of the Monocyte WBC has a kidney- or bean-shape. Lymphocyte’s nucleus has a round uniform shape in case of normal blood sample (Fig 9(B)) or a non-uniform oval-shape for cancer blood sample (Fig 9(D)). It is worth mentioning that, the nucleus shape and size of Lymphocyte WBCs are varying in case of blood cancer.

### 5.2 Optical reconstruction results

**Fig 10:**
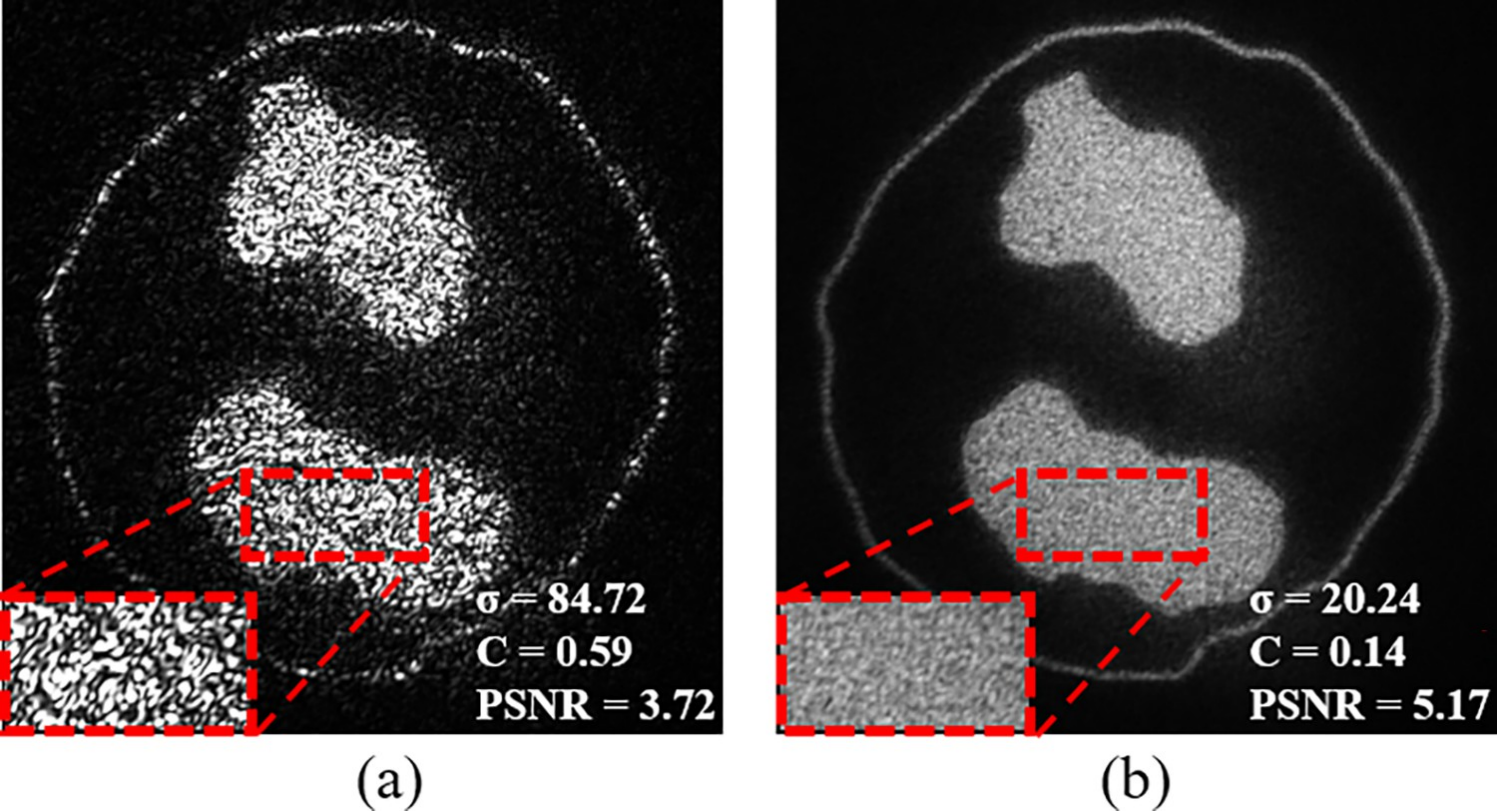
The optical reconstruction of Eosinophil WBC (a) before and (b) after applying the speckle noise reduction algorithm. Where σ is the standard deviation; C is the speckle noise contrast; PSNR is the peak signal to noise ratio.

As an example, for evaluating the utilized speckle noise reduction algorithm, Eosinophil WBC reference image is used. Fig 10(A) and 10(B) show the optical reconstruction of Eosinophil WBC image before and after applying the speckle noise reduction algorithm. The results exhibit that, σ and C are decreased after applying the speckle noise reduction algorithm, from 84.72 and 0.59 to 20.24 and 0.14, respectively, and PSNR is increased from 3.72 to 5.17. This indicates an improvement of the optical reconstructed results after applying the speckle noise reduction algorithm.

**Fig 11:**
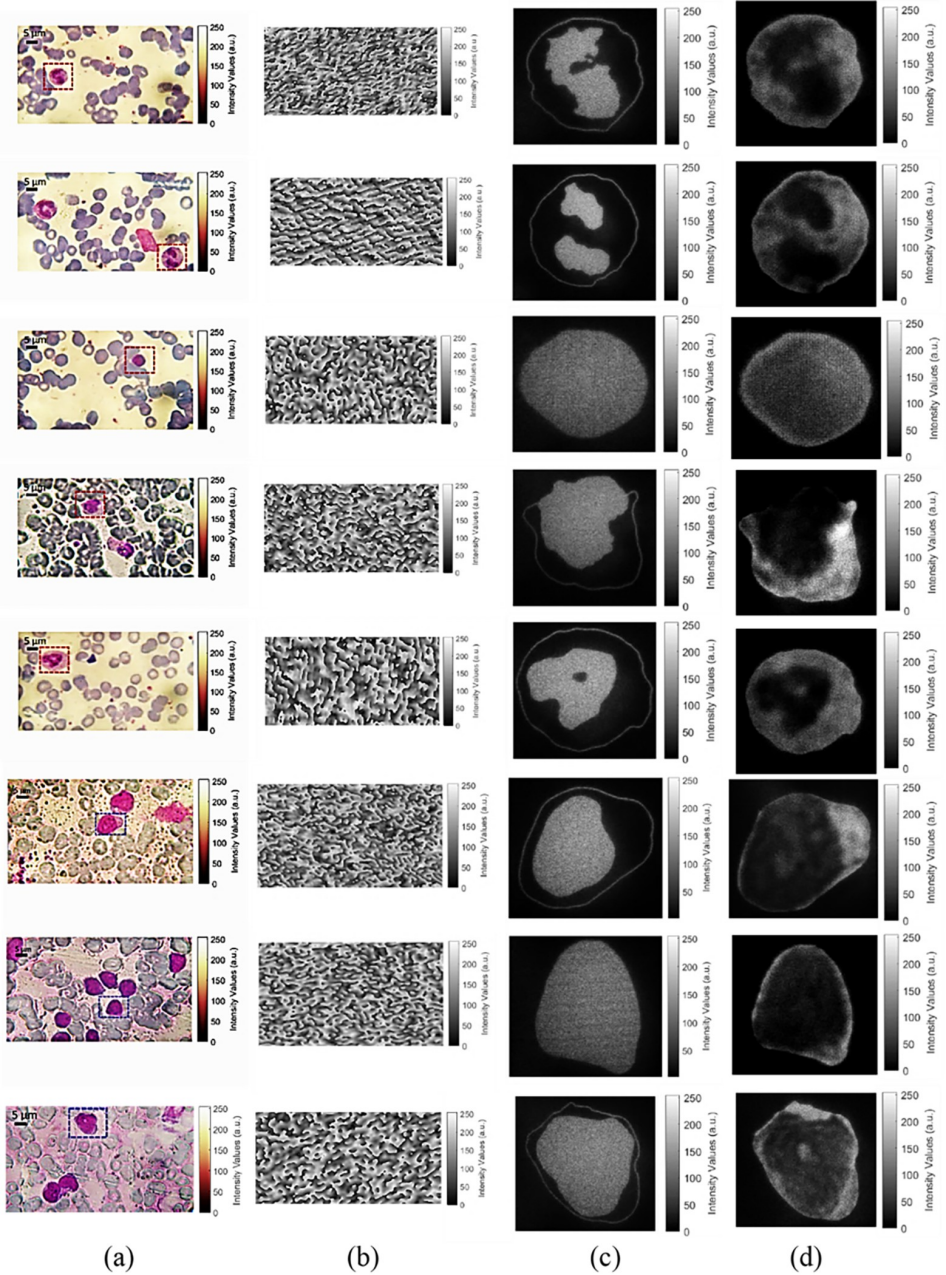
(a) Microscope colored raw images of normal WBCs (marked with red dashed box) and cancer WBCs (marked with blue dashed boxes). (b) The calculated CGHs of the morphology of the targeted WBCs (in the dashed boxes). (c) The optical reconstruction of the WBCs morphology. (d) The optical reconstruction of the WBCs structure.

The optical reconstruction results after applying the speckle noise reduction algorithm for normal and cancer WBCs are demonstrated in Fig 11. Where the cell that is surrounded by a red box is a normal cell, while the cell that is surrounded by a blue box is a cancer one, both types of cells are shown in Fig 11(A). The calculated CGHs of the morphology of the targeted WBCs are presented in Fig 11(B). The phase-only CGHs values vary from 0 to 255, and they are displayed on the phase-only SLM for optical reconstruction. The optical reconstruction of cells morphology and structure are presented in Fig 11(C) and 11(D).

From the optical reconstruction results of the cells morphology (Fig 11(C)), the nucleus is seen clearly and the cells boundaries are clear as well. Therefore, some useful characteristics can be measured quantitively for each kind of cells, these characteristics are demonstrated in Table 1. It shows the length of major and minor axes, nucleus area, and whole cell area. As mentioned before, for specific disease (i.e. cancer), the number of Lymphocyte cells noticeably increase. Besides, it was found that the measured cells characteristics are changed as well. The major axis length, minor axis length, and cell area were 6.94 μm, 6.48 μm, and 35.19 μm^2^, for a Lymphocyte cell of the normal blood sample. Whereas, for three different Lymphocyte WBCs of the cancer blood sample, it was found that the major and the minor axes of the cells are increased by an average 41.69%, 33.02% more than a normal Lymphocyte cell. Moreover, the nucleus areas for the three Lymphocyte cells of the cancer sample are increased by 187.16%, 67.97%, 125.04% more than the nucleus area of the normal Lymphocyte cell. On the other hand, from the optical reconstruction of cells structure (shown in Fig 11(D)), the granules that are containing the proteins are clearly seen in the optical reconstruction of Basophil, Eosinophil, and Neutrophil WBCs. Lymphocytes and monocytes are non-granulocytes, this is seen clearly from the optical reconstruction results (shown in Fig 11(D)).

**Table 1:** The measured characteristics of the normal and the cancer WBCs.

Cell type	Major axis length of the cell (μm)	Minor axis length of the cell (μm)	Nucleus area (μm^2^)	Cell area (μm^2^)
**Normal WBCs**				
**Basophil**	10.22	9.57	29.01	76.60
**Eosinophil**	12.76	11.74	31.72	117.48
**Lymphocyte**	6.94	6.48	35.19	35.19
**Monocyte**	12.00	9.66	84.39	89.18
**Neutrophil**	16.26	11.15	50.70	141.86
**Cancer WBCs**				
**Lymphocyte cell 1**	9.23	7.71	49.83	101.05
**Lymphocyte cell 2**	9.17	8.46	59.11	59.11
**Lymphocyte cell 3**	11.10	9.68	73.72	79.19

**Fig 12:**
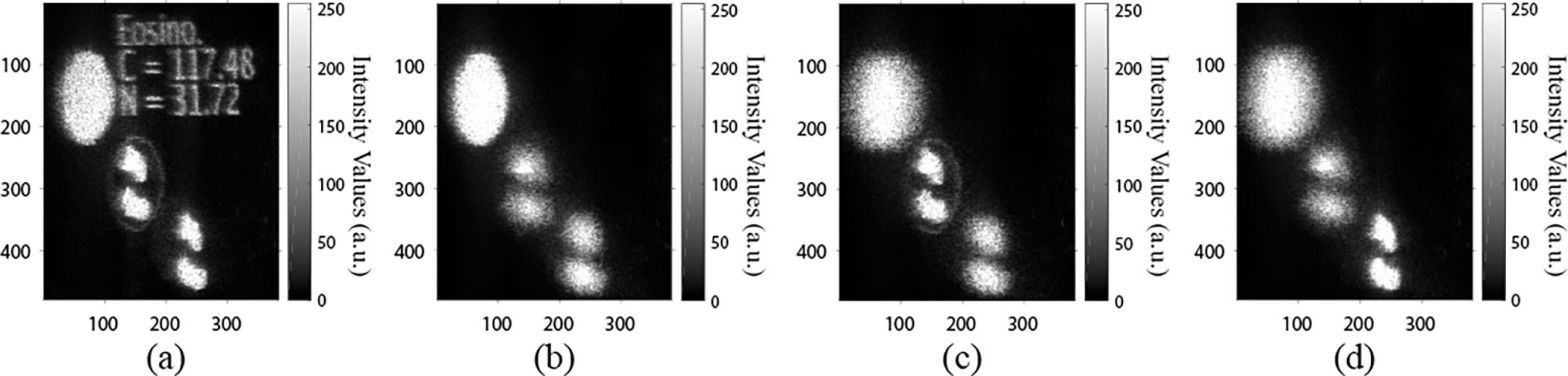
The 3D optical reconstruction of the Eosinophil cell from a single 2D hologram: (a) all structures are focused, (b) the cell is focused, (c) the edges of the cell and its nucleus are focused, and (d) the nucleus only is focused.

Fig 12 provides a 3D visualization map of the Eosinophil cell (as an example), the edges of the using a single 2D hologram. As additional information, we include the name of the visualized cell (Eosinophil), its area (C in μm), and the area of its nucleus (N in μm) in the optical reconstruction as demonstrated in Fig 12(A). Fig 12(B)–12(D) show the feasibility of the proposed method for the optical segmentation and discrimination of a specific feature (e.g., size or shape) based on the hologram modulation (described in the previous subsection 3.1). As observed in Fig 12(B), the cell body is discriminated and selected in the optical reconstruction to be focused and the other structures are out of focus. In Fig 12(C), the edges of the cell and its nucleus is discriminated and focused and the other structures are out of focus. The nucleus only can be selected and focused as depicted in Fig 12(D).

**Fig 13:**
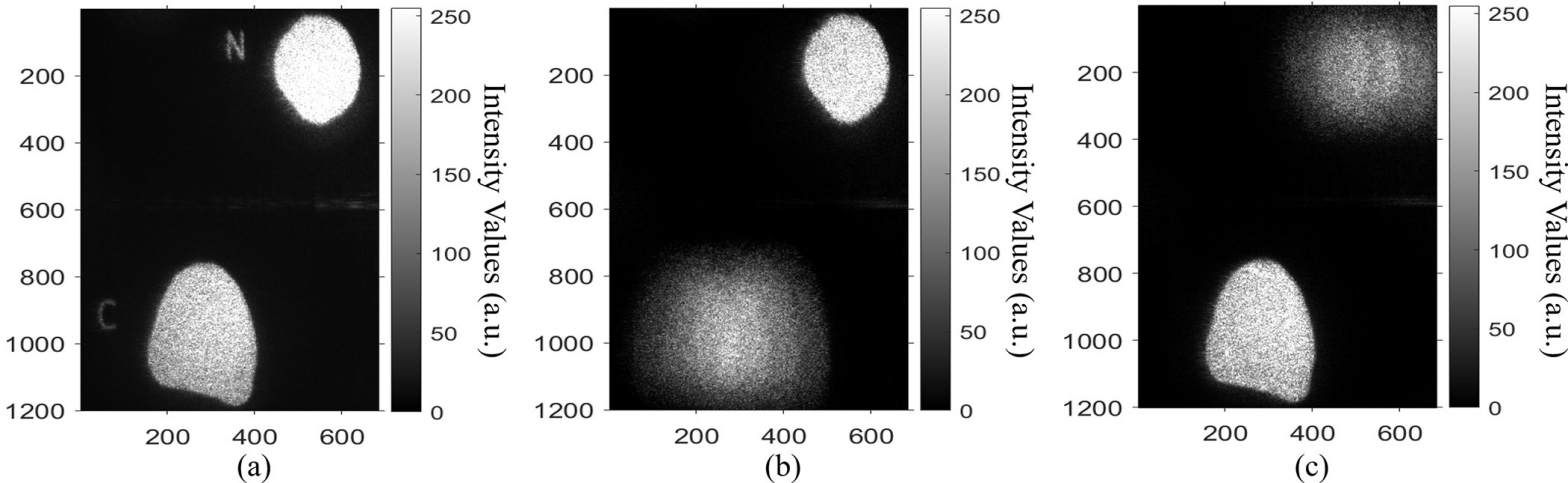
Discrimination between normal cell (N) and cancer cell (C) using a single 2D hologram (a) the two cells are focused, (b) the normal cell is focused and the cancer cell is out of focus, and (c) the cancer cell is focused and the normal cell is out of focus.

Additionally, the optical reconstruction of a single 2D hologram shows the ability to discriminate two WBCs (normal and cancer) as depicted in Fig 13. As an additional information, the cell type (i.e., N for normal WBC and C for cancer WBC) was included in the optical reconstruction for easier discrimination of the two types of cells. The 3D optical reconstruction of the two WBCs is shown in Fig 13(B) and 13(C). Fig 13(B) displays the normal cell (N) focused and the cancer cell (C) out of focus, while Fig 13(C) displays the cancer cell (C) focused and the normal cell (N) out of focus. Therefore, this display method can be used for training hematologists for discriminating normal and cancer WBCs using only a single 2D hologram.

Such a display method can help untrained hematologist for better understanding of the morphological structures of WBCs and getting fast information about its characterization parameters in real-time. The calculation of CGH is based on fast Fourier transform and its implementation using Matlab requires *Mlog*_2_*M* floating point operations to compute for an image of a size of *M*×*M*, where *M* represents the number of rows and columns. In each iteration of an IFTA, four F were implemented. Based on the power of the utilized graphics processing unit (GPU) / central processing unit (CPU), the time required to reach the convergence of the algorithm has been calculated. In the current study, the convergence time of the algorithm was 65.812 sec using Core i7-1165G7 CPU 2.8 GHz with 32 GB RAM, MATLAB 2018a. This time could be significantly decreased in milliseconds if the algorithm is parallelly computed with the GPU. For that reason, the overall process of the holographic projection (i.e., CGHs calculation and the optical reconstruction) is considered as a real-time process. Unlike our proposed discrimination method, complexity and time-consuming processing are the main features of the common discrimination methods such as pattern recognition. On the other hand, the use of the conventional bright-field microscopic images can not provide detailed information of the cell and its internal structure. For instance, the diagnosis of a specific blood disease is subjected to inter-observer variability.

## 6- Conclusions

In this paper, a holographic projection system for discrimination between normal and cancer white blood cells (WBCs) of human blood cells was proposed. The WBCs are precisely segmented from the optical microscope images using a developed in-house MATLAB code. The proposed segmentation method was evaluated by estimating the optimum threshold value to segment the WBC from the optical microscope image and by determining the execution time of the segmentation process. It was found that a threshold value of 70% of the bright intensity (*I*_*B*_) satisfies most of the test cases and the average execution time of the segmentation process was 5.35 sec. Then, the computer-generated holograms (CGHs) of the segmented WBCs are calculated and optimized using Iterative Fourier transform algorithm (IFTA). The designed phase holograms were evaluated using the root mean square error (RMSE) which was used to monitor the quality of the numerical reconstructed images, and to determine the optimal number of iterations which is based on a suggested stopping criterion. The optimal number of iterations was 60 for an extended phase-hologram of size 7680 × 4320 pixels, with calculation time 70.321 sec using Intel Core i7-3210 CPU 2.5 GHz with 4 GB RAM. However, this time could be significantly decreased in milliseconds range if the approach was paralyzed with a graphical processing unit (GPU). Within the optical reconstruction, temporal multiplexing of spatial frequencies was applied to suppress the speckle noise and to get improved reconstructed images. The calculated phase holograms are uploaded on a reflective phase-only spatial light modulator (SLM) for optical reconstruction. The whole process of displaying the phase holograms and capturing the reconstructed images took less than 1 sec, since the switching time of the used SLM was 50 msec. The optical reconstruction results were evaluated using the scaled signal to noise ratio (SNR), standard deviation (σ), and the speckle contrast (C). The obtained results revealed a significant quality improvement of the optically reconstructed images after applying the speckle noise reduction algorithm. The experimental results exhibit that the proposed holographic projection system can be used as an effective optical method for visualizing and discriminating normal and cancer WBCs. Moreover, it is capable of providing a 3D visualization map of one cell or a segmented structure optically. Furthermore, this optical display method is considered as a valuable tool in the clinical trials that can help untrained hematologist for better understanding of WBCs morphology (shape and size) and getting fast information about its characterization parameters. Therefore, the diagnosis and discrimination between cancer and normal WBCs could be rapidly and accurately achieved with detailed information which couldn’t be obtained by the conventional bright-field microscopic images only.

## Supporting information

S1 Fig (BMP)

S2 Fig (BMP)

S3 Fig (BMP)
