# Supplementary figures and images for: Discrimination between normal and cancer white blood cells using holographic projection technique
Source: PLoS One. 2022 Oct 20;17(10):e0276239. doi: 10.1371/journal.pone.0276239 (PMC9584458; doi:10.1371/journal.pone.0276239)

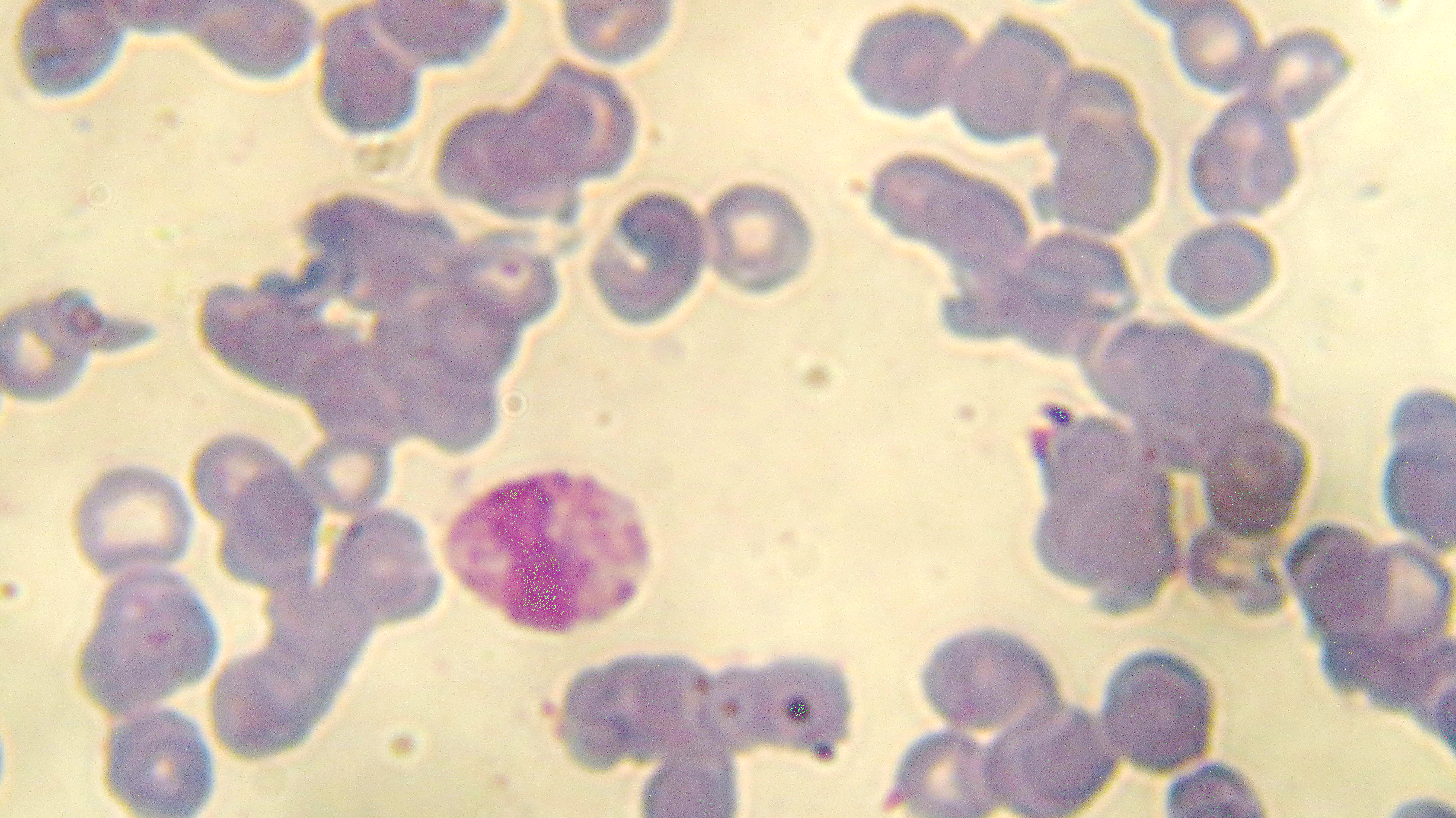

Supplement: S1 Fig — (BMP) [file pone.0276239.s001.bmp]

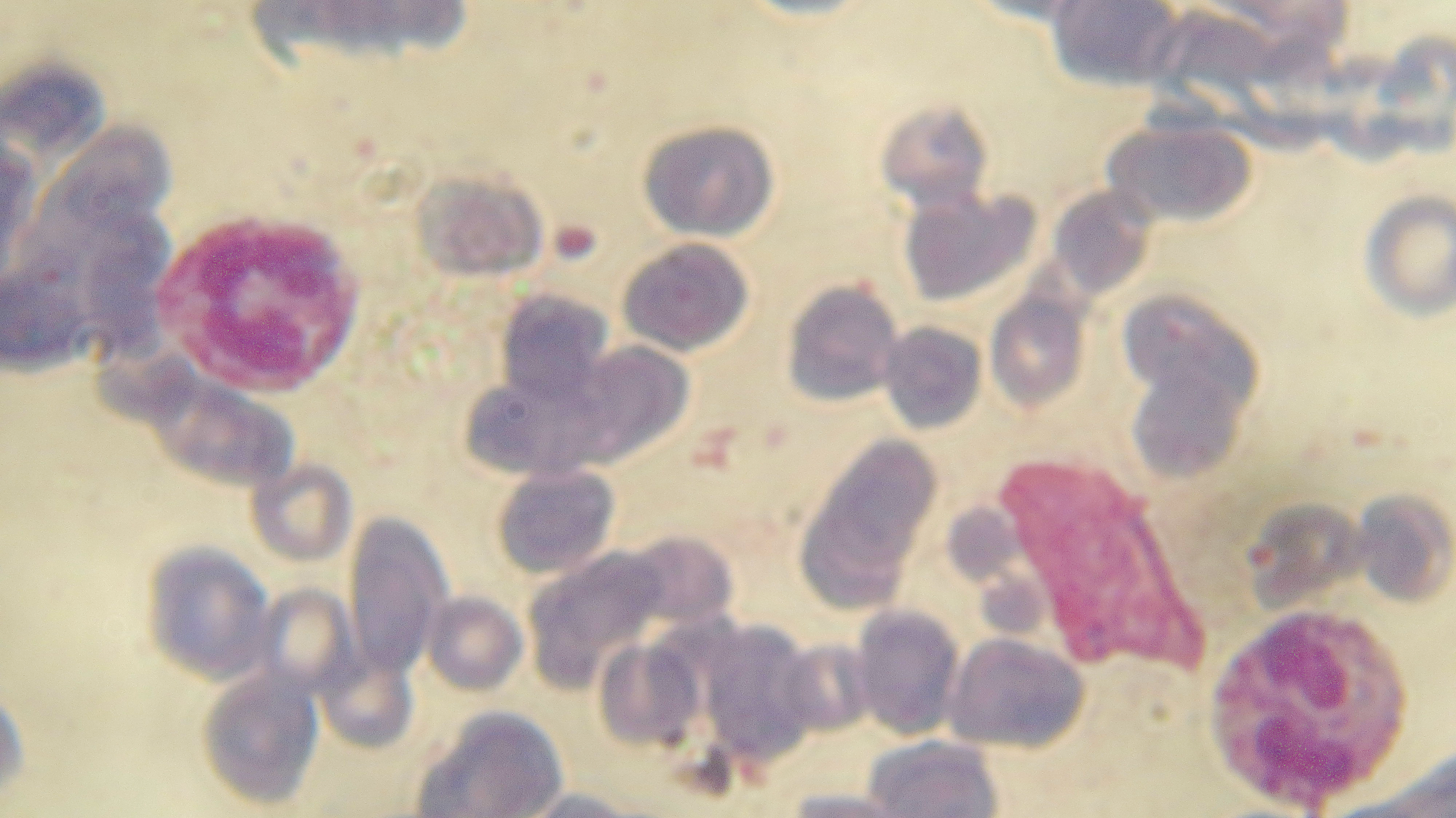

Supplement: S2 Fig — (BMP) [file pone.0276239.s002.bmp]

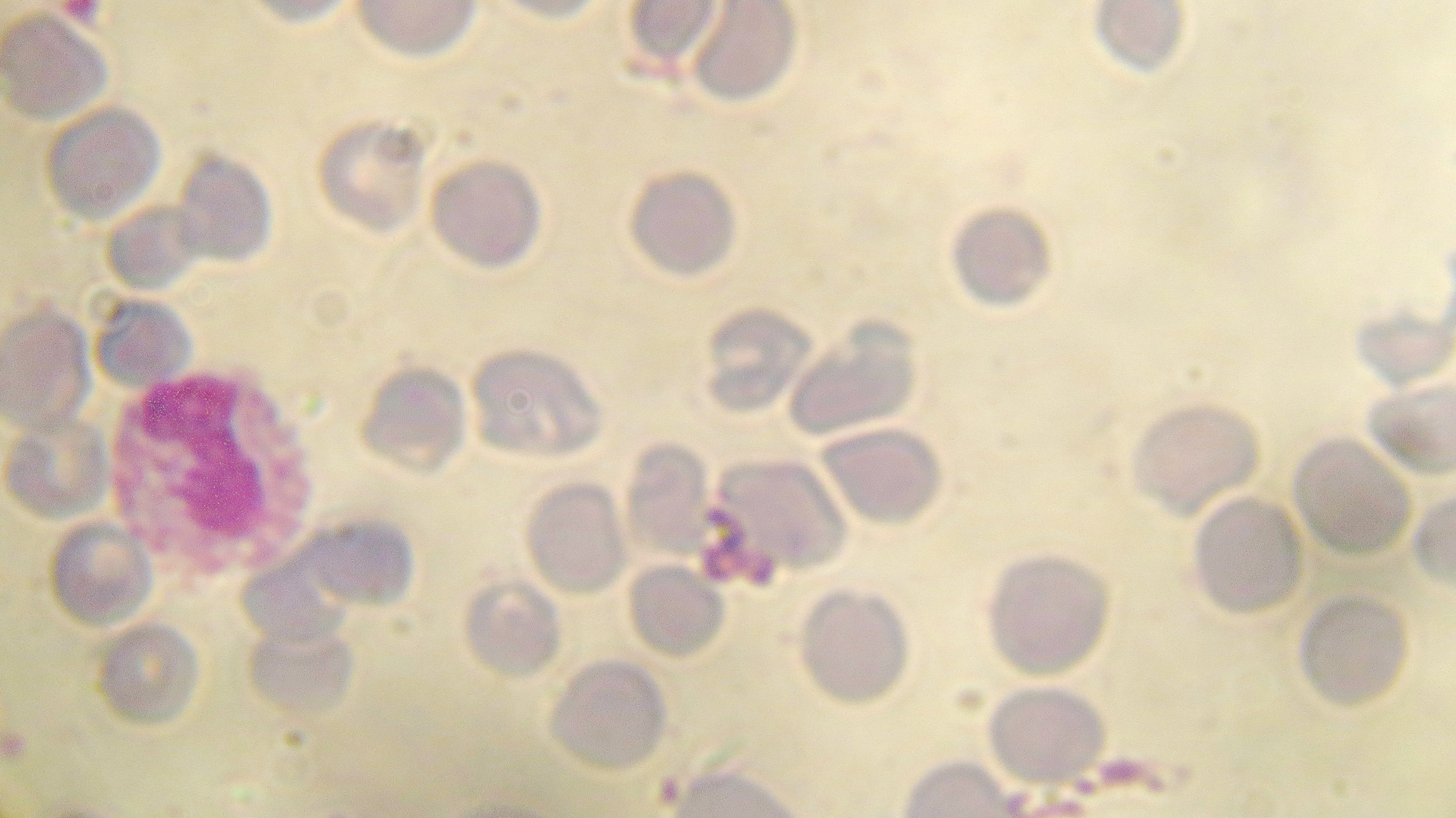

Supplement: S3 Fig — (BMP) [file pone.0276239.s003.bmp]
